# Predictive Roles of Thinking Styles in Coping Strategies Among Mainland Postgraduate Students in Hong Kong

**DOI:** 10.3389/fpsyg.2021.693637

**Published:** 2022-01-28

**Authors:** Siyao Chen

**Affiliations:** Faculty of Education, University of Hong Kong, Pokfulam, Hong Kong SAR, China

**Keywords:** thinking styles, coping strategies, gender role orientations, postgraduate students, Hong Kong

## Abstract

The primary objective of the present research was to explore the statistical predictive power of thinking styles in coping strategies beyond demographic factors. One hundred and forty-eight mainland postgraduate students were administered to the Thinking Styles Inventory-Revised II (TSI-R2) and the Coping Orientation to Problems Experienced (COPE) Revised. Results indicated that Type I thinking styles primarily predicted adaptive coping strategies, while Type II thinking styles positively contributed to maladaptive coping strategies. Results in the present research were largely in the expected directions beyond the influence of demographic factors. Furthermore, thinking styles varied as a function of age, gender, gender-role orientation, and marital status. Implications for postgraduate students, academics, university administrators, and the limitations of the research, are discussed.

## Introduction

With the internationalization of higher education, a growing number of mainland postgraduate students have been attracted to Hong Kong and taken up the largest proportion of non-local students. For example, in 2019–2020, there were 6,964 non-local postgraduate students, accounting for 61.9% of registered postgraduate students in eight government-funded universities in Hong Kong. Of non-local postgraduate students, 5,831 mainland postgraduate students occupied the largest proportion of 83.5% (University Grants Committee, 2020). Thus, the group of mainland postgraduate students in Hong Kong deserves much attention. However, previous studies reported that these students face high levels of stressors in fields of acculturation (Bhowmik et al., 2018), academic sojourn (Brown and Holloway, 2008), and psychological stress (Yu et al., 2019). These are harmful to their emotional, psychosocial, psychological aspects, and academic performance (Demes and Geeraert, 2015; Tummala-Narra et al., 2016). Therefore, to cope with these stressful situations, coping strategies are of great significance to these specific cohorts of students.

Intellectual styles, preferred ways of performing tasks or processing information (Zhang and Sternberg, 2005), have been statistically proven to be significant in both academic and non-academic settings (Zhang, 2017). As the most general and comprehensive model, thinking styles served as representatives of intellectual styles. Firstly, thinking styles are one of the most comprehensive style constructs. This style construct identifies an individual's styles in five dimensions and covers all three traditions in the field of intellectual styles (Sternberg, 1997). Secondly, thinking styles are the original and theoretical foundations of intellectual styles, which help to better understand the theory of intellectual styles. Coping strategies are conceptualized as the reactions of individuals to stress conditions related to potentially negative consequences (Folkman and Lazarus, 1984). Previous empirical studies have revealed the relationships between coping strategies and other variables in aspects of personal factors (Lue et al., 2010), environmental factors, and other individual-difference variables. Nevertheless, according to the last category, limited studies have focused on examining factors influencing coping strategies in intellectual styles (Gras et al., 2009; Yuan et al., 2017). Findings on their relationships enrich the empirical evidence on both two constructs. Furthermore, previous studies have also proved the value of thinking styles in individual-difference variables. Firstly, adaptive Type I thinking styles have been widely proved to be correlated with positive individual variables. For instance, Type I thinking styles are positively linked to future time perspectives, a mature defense style, academic performance, and higher levels of cognitive development (Zhang, 2002; Karagiannopoulou and Christodoulides, 2005; Kuan and Zhang, 2020). Secondly, thinking styles also exhibited predicting powers in individual-difference variables. For instance, Type I thinking styles play predicting roles in academic performance, critical thinking dispositions, and sense of purposefulness (Zhang, 2002, 2003, 2004). However, there are also controversies over relationships between thinking styles and some other variables. For instance, inconsistent findings have been yielded over the relationships between thinking styles and coping strategies (Appelhans and Schmeck, 2002; Gras et al., 2009). The statistical predictions of thinking styles in coping strategies also remained under-explored. Therefore, to fill in these gaps, the present study aimed to examine the predicting roles of thinking styles in coping strategies among mainland postgraduate students in Hong Kong.

Demographic factors (i.e., age, gender, marital status, and socioeconomic status) can be considered as one of the significant determinants influencing thinking styles. However, controversies over the influences of age on thinking styles have been detected (Zhang and He, 2011; Al-Thani et al., 2014; Kuan and Zhang, 2020). Similarly, different perspectives have also been made on the functions of gender on thinking styles (Witkin and Berry, 1975; Balkis and Isiker, 2005; Gridley, 2006). Some scholars believed that gender exerts impacts on thinking styles (Balkis and Isiker, 2005; Zhang and Sternberg, 2006), while others contended that no gender difference exists in thinking styles (Grigorenko and Sternberg, 1997; Gridley, 2006). The latter view can be accounted for by social role orientations. Men and women are brought up with different social expectations (Vaught, 1965) and attitudes (Maccoby and Jacklin, 1974), which may lead to different preferences for thinking styles. Nevertheless, some women who are raised and possess past experiences similar to those more typical of men might possess a masculine self-concept (Bem and Lenney, 1976). Thus, those women might possess similar thinking styles to men. Nevertheless, few studies simultaneously consider both social role orientations and their gender in relationships with thinking styles. Marital status is significant to postgraduate students (Poyrazli and Kavanaugh, 2006) and is closely associated with thinking styles. Both constructs have their own relationships with career ambition, social adjustment strain, perceived stress, social support, motivations, self-efficacy, self-esteem, and well-being (Poyrazli and Kavanaugh, 2006; Fan, 2016). However, the comparison of married and unmarried postgraduate students in the direct relationship of thinking styles is still under-explored. Socioeconomic status has been statistically proven to be significant in postgraduate study (Watson et al., 2010; Han et al., 2014) and is one of the factors influencing thinking styles (Alevriadou et al., 2004). Besides, disciplines also show inevitable influences on thinking styles due to unique identities and the nature of various academics (Becher, 1981; Pettigrew and King, 1993). Furthermore, contradictory findings have also been found in the thinking styles among students of social science (Balkis and Isiker, 2005; Kim, 2010). Some specific style constructs have not been explicitly explained in relation to different academic disciplines. Therefore, demographic factors (i.e., age, gender, gender-role orientations, marital status, and socioeconomic status) are taken into consideration in the relationship with thinking styles among mainland postgraduate students in Hong Kong.

This study contributed to the advancement of research on both the influential role of demographic factors on thinking styles and the predictive roles of thinking styles for coping strategies. Theoretically, the present study can give insight to understand the relationships between these two constructs, respectively. Practically, the results of this study may provide implications for individual development in higher education by developing Type I thinking styles or adaptive coping strategies.

## Coping Strategies and Three-Dimensional Coping Strategy Model

The conceptual framework of coping strategies was initially proposed by Folkman and Lazarus (1984). They referred to coping strategies as reactions of individuals by changing cognitive and behavioral efforts to stress conditions related to potentially negative consequences (Folkman and Lazarus, 1984). Lazarus argued that stress consists of three processes, and coping is the last process in the stress response (Carver et al., 1989). Scholars generally categorized coping scales, such as problem-focused and emotional-focused coping (Folkman and Lazarus, 1980), dual-process models (Compas et al., 1997; Skinner, 1999), and hierarchical models of coping (Ryan-Wenger, 1992). Despite the vague classification (Skinner et al., 2003) and dysfunctional homogeneous (Skinner et al., 2003) of some coping strategies, a three-dimensional coping strategy model (Yuan et al., 2017) rather than Brief COPE (a short version of COPE, Carver, 1997) is proposed to examine coping strategies and divide them into three hierarchical types. The first category is self-directed coping, which is defined as an individual taking advantage of his/her behavioral and cognitive efforts in dealing with stress. This type includes active coping, positive reframing, and planning. The second category is other-directed coping, which is conceptualized as the preference of an individual for turning to others (people or things) for help when experiencing stress. This type pertains to venting, using emotional support, using of instrumental support, and self-distraction. The last category is relinquished control coping, which is characterized as the absence of an effort to deal with stressful situations (Weisz et al., 1994). This type consists of social withdrawal, acceptance, and self-blame. In this model, self-directed coping is associated with the nature of adaptivity, and relinquished control coping is related to maladaptive values. However, there are some controversies over the value of other-directed coping. While a sample of 646 secondary students in China suggested that other-directed coping strategies are maladaptive (Yuan et al., 2017), Carver et al. (1989) compared other-directed coping strategies as a double-edged sword. Specifically, the use of social-emotional support (one scale in other-directed coping strategies) can be adaptive when more support and encouragement fostering coping, nevertheless, it can also be maladaptive when it becomes a source of sympathy and a way to simply vent one's feelings (Billings and Moos, 1984; Tolor and Fehon, 1987). What should be noted is that the use of adaptive coping or maladaptive coping can be influenced by the participants (Roth and Cohen, 1986).

## Three-Fold Model of Intellectual Styles and Thinking Styles

The term “style” was initially introduced to psychology by Allport (1937). In the history of the field of styles, cognitive styles appeared earlier than other notions such as learning styles and thinking styles due to the necessity for the connection between cognition and personality, and the “cognitive-styles movement” proliferated in the late 1950s and flourished for more than two decades (Adorno et al., 2019). However, the proliferation waned due to the plethora of existing style constructs and the resurgent interest of scholars in the discipline of education in the 1970s. In the mid-1980s, the field of styles began to arouse scholarly interest again because the theory of abilities and personality could not fully explain individual differences. However, lacking a conceptual framework (Coffield et al., 2004) and with little understanding of the relationships between intellectual styles and other psychological constructs, the style field attempted to integrate various style constructs in the past four decades.

Four integrated models have been introduced: Curry's (1983) model, Miller's (1987) model, Grigorenko and Sternberg's (1995) model, and Zhang and Sternberg's (2005) three-fold model. The first two models have been widely criticized for being unable to provide sufficient construct reliability and validity (Curry, 1983; Messick, 1994; Zhang and Sternberg, 2006). In Grigorenko and Sternberg's (1995) model of style traditions, the deficiency is that the theory may suffer from a lack of solid theoretical foundation, and overall measurement models are inconsistent with the theoretical models. Therefore, factor structures are not efficient to yield empirical evidence (Ross, 1962; Keller and Holland, 1978; Joniak and Isaksen, 1988).

Considerable endeavors have been made by Zhang and Sternberg (2005) in integrating a variety of style constructs and conceptualizing the notion of the three-fold model of intellectual styles. The notion of intellectual styles, a term encompassing ten influential existing style constructs (Zhang, 2017), is conceptualized as preferred ways of doing tasks or processing information (Zhang and Sternberg, 2005). Zhang and Sternberg's (2005) three-fold model of intellectual styles has been proposed to resolve the three most controversial issues of intellectual styles (i.e., style value, style malleability, and style overlap) by presenting empirical evidence within a unified scientific framework and integrating all these style constructs into three types. Creativity-generating Type I intellectual styles represent low degrees of structure, cognitive complexity, autonomy, and norm-conforming. Type II intellectual styles denote high degrees of structure, cognitive simplicity, conformity, and authority. Type III intellectual styles are largely differentiated depending on situations, such as demands of a specific task and individual's level of interest in the task (Zhang and Sternberg, 2005, p. 36).

The nature of thinking styles should be clearly stated (i.e., style value, style malleability, and style overlapping). Concerning the style value, Zhang and Sternberg (2006) state that the majority of style constructs are value-laden rather than value-free. That is, Type I intellectual styles are generally superior to Type II intellectual styles since Type I are generally linked to more adaptive attributes (Zhang, 2004; Zhang and Jing, 2014) and more desirable developmental outcomes (Broberg and Moran, 1988; Guisande et al., 2007). In addition, Type III intellectual styles are neither good nor bad. Concerning style malleability, style constructs are fluid states instead of fixed traits. Although some style constructs may be relatively stable, they still can be malleable over a longer period of time. Concerning the last issue, while various style constructs empirically overlap, they still keep their uniqueness.

The theory of mental self-government (Sternberg, 1988, 1997) is the most updated and general model in Zhang and Sternberg's (2005) three-fold model of intellectual styles. Metaphorically, Sternberg (1988) compared multiple ways people managed their own activities to a variety of ways the government adopted to manage society. These various methods are thinking styles (also called the theory of mental self-government), which refer to the preferred and comfortable ways that people self-govern or deal with tasks (Sternberg, 1988, 1997). He suggested 13 thinking styles falling along five dimensions, those being functions, forms, levels, scopes, and leanings. In the three-fold model, these 13 styles were further operationalized into three types: Type I (e.g., the legislative, judicial, global, hierarchical, and liberal styles), Type II (e.g., the executive, local, monarchic, and conservative styles), and Type III (e.g., the oligarchic, anarchic, internal, and external styles) thinking styles.

The validity of Sternberg's theory of mental self-government has been frequently operationalized by the Thinking Styles Inventory Revised II (TSI-R2, Sternberg et al., 2007), which was the updated version of the TSI-R (Sternberg et al., 2003) to further improve the internal consistency for the anarchic style. This updated inventory has been practiced extensively in many studies in several cultures, including the United States (e.g., Bishop and Foster, 2011), South Africa (e.g., Murphy and Janeke, 2009), Turkey (e.g., Fer, 2007), China (e.g., Zhang, 2010), and the United Kingdom (Zhang and Higgins, 2008). The empirical evidence of thinking styles focuses on the influential factors (i.e., the personal factors and environmental factors) on thinking styles and the role of thinking styles in other attributes. However, the relationships between coping strategies and thinking styles and the relationships between demographic factors and thinking styles have not been sufficiently explored.

## Demographic Factors and Their Relationships With Thinking Styles

One of the natures of thinking styles is style malleability, which is defined as whether styles can be malleable or modified due to outside forces or remain static over time (Armor and Taylor, 2003). Zhang (2017) demonstrated that intellectual styles that include thinking styles can be modified or malleable by socialization. Since the socialization effects of demographics, such as age, gender, and gender-role orientation (Carlson and Levy, 1968; Hofstede, 1980), have been much discussed and indicated in previous literature, the relationships between thinking styles and demographic factors are needed to be considered in the present research.

Empirical evidence on age difference in thinking styles is inconsistent (Zhang and He, 2011; Al-Thani et al., 2014; Kuan and Zhang, 2020). These inconsistent findings can be partially accounted for by cultural differences, teaching methods (Lau, 2014), instructional modes (Fan, 2012), or interactional effects of age and gender in the role of thinking styles. Besides, the changeability of intellectual styles might be repeated and bidirectional over different periods of durations. For example, based on the data collected from Kuan and Zhang (2020), the authors found that older participants are positively associated with Type II thinking styles (e.g., higher executive styles) in secondary schools. Furthermore, data gathered from a sample of university students, Zhang and He (2011) revealed that older students tended to score significantly higher in the frequency of using Type I thinking styles. Similarly, this phenomenon has also been found in other constructs (e.g., field dependence/independence) of the family of intellectual styles. Since young adulthood (aged from 17 to 24) might be a division of the development of field dependence/independence (Zhang, 2013), the higher extent of field independence might be linked to the increase of age before young adulthood. Conversely, the higher extent of field dependence is related to older people after young adulthood. The division between traditional-aged and non-traditional-aged postgraduate students overlaps with young adulthood, there is a need to consider the age difference in thinking styles in the present study.

Inconsistent findings are also yielded over whether gender difference influences thinking styles (Witkin and Berry, 1975; Balkis and Isiker, 2005; Gridley, 2006). In previous studies, a general pattern of gender difference in thinking styles is that men tended to score higher on the majority of Type I thinking styles and women scored higher on most of the Type II thinking styles (Zhang and Sternberg, 2006). This phenomenon can be accounted for by the effect of gender-role orientation (Balkis and Isiker, 2005; Zhang and Sternberg, 2006). Traditionally, the stereotypical view is that men should be the dominant ones who make rules and big decisions in the family and focus on the bigger picture. On the other hand, women should be obedient, follow rules, make minor decisions, and paycareful attention to details. Besides, women tended to score higher on the hierarchical style because if they are in pursuit of success, they need to fulfill multiple roles within their family and work (Pinker, 1997). Thus, the gender difference in thinking styles may be partly due to social factors, such as different social expectations (Stuart et al., 1965; Vaught, 1965), gender-role stereotypes (Nash, 1979), and attitudes (Maccoby and Jacklin, 1974; Huston, 1985).

Previous findings revealed no significant gender difference in thinking styles (Grigorenko and Sternberg, 1997; Gridley, 2006). Nevertheless, gender differences exist in thinking styles. This phenomenon can also be accounted for by social-role orientation. As aforementioned, men and women are brought up with different social expectations, which may lead to different preferences for thinking styles. Nevertheless, some women who are raised and have past experiences more similar to a typical male socialization experience might possess a masculine self-concept (Bem and Lenney, 1976). For example, in intellectual styles (e.g., perceptual styles), stereotypically masculine individuals were more field independent than those who were more stereotypically feminine (Zhang, 2013). However, few previous studies simultaneously consider both social role orientations and their gender in relationships with thinking styles.

Studies on the marital status among postgraduate students mostly focus on married female candidates, marital satisfaction (Powers et al., 2004), and mental health. To be specific, those studies specifically focused on their career ambition, social adjustment strain, perceived stress, social support, motivations, self-efficacy, self-esteem, and well-being (Poyrazli and Kavanaugh, 2006; Fan, 2016). While these factors are associated with thinking styles, marital status needs to be considered in thinking styles among postgraduate students.

Socioeconomic status has been investigated in the field of postgraduate study, such as metacognitive awareness (Jain et al., 2018), self-efficacy (Han et al., 2014), and occupational aspiration (Watson et al., 2010). Socioeconomic status can be considered as one of the factors influencing thinking styles. For example, higher socioeconomic status is associated with specific creativity-generating Type I thinking styles (Grigorenko and Sternberg, 1995; Zhang and Postiglione, 2001). One explanation is that children from upper/middle socioeconomic status cognitively develop due to the stimuli they receive within their family environments (Alevriadou et al., 2004). Zhang and Sternberg (2006) further explained that students from higher socioeconomic families have more chances to be challenged by different, unpredictable situations. By overcoming these challenges, they can be more creative and form a higher form of complexity. Thus, it is necessary to take socioeconomic status into account when considering the relationship with thinking styles among postgraduate students.

Different academic disciplines possess unique identities, which might lead to different thinking styles (Becher, 1981). To elaborate, according to the nature of different disciplines, the ways of supervising and training postgraduate students can be varied due to some factors, such as small-group discussions (Ho, 2011), educational experiences, and tacit knowledge (Zarshenas et al., 2014).

Vice versa, the influence of thinking styles can also be perceived in varying academic disciplines. While students of different thinking styles may choose different disciplines, the difference in thinking styles might still exist since those who find themselves incompatible with the learning environment would withdraw from the program and adapt to new environments (Witkin and Berry, 1975). This invisible shifting population can be seen as a piece of underlying evidence for the relationships between disciplines and thinking styles. Thus, different thinking styles in different disciplines can be seen as a result of the nature of different disciplines (Pettigrew and King, 1993), different abilities, and required objectives, which might push learners in a certain direction.

Studies focusing on the differences between thinking styles and specific academic disciplines of students yielded the following findings (Lam, 2000; Balkis and Isiker, 2005; Kim, 2010). Students of natural science prefer the global (I), executive (II), and internal (III) styles. And students of social science tend to deal with tasks with the liberal (I), conservative (II), monarchic (II), and external (III) styles. Finally, students of humanities prefer conservative (II), internal (III), and oligarchic styles (III). However, according to students of social science, their preference for the liberal style found by Kim (2010) is contradicted by the conservative style contended by Balkis and Isiker (2005). Furthermore, some style constructs, such as the legislative (I), judicial (I), hierarchical (I), anarchic (III), and local (II) styles have not been explicitly explained concerning students of different academic disciplines. In the present study, social science, natural science, and humanities as the general classification of disciplines to examine the relationships between different academic disciplines and thinking styles among postgraduate students.

## Coping Strategies and Their Relationships With Thinking Styles

Conceptually, thinking styles are the preferred ways of dealing with tasks (Sternberg, 1988), and coping strategies are referred to as the preferred ways of coping with stress (McCrae, 1982). In terms of the issue of value, both thinking styles and coping strategies are also value-laden or value-differentiated rather than value-free (Zhang and Sternberg, 2005). Empirically, inconsistent findings are yielded from the relationships between intellectual styles and coping strategies. Appelhans and Schmeck (2002) suggested that Type I intellectual styles (e.g., deep-thinking learning strategies) are related to adaptive stress-coping strategies, and Type II intellectual styles (e.g., memorization learning strategy) are associated with maladaptive avoidance coping. On the other hand, Young (2005) contends that Type II intellectual styles (e.g., impulsivity) are associated with both adaptive (e.g., the confrontative strategy) and maladaptive coping strategies (e.g., the escape-avoidance strategy). Similarly, Gras et al. (2009) also found that Type I intellectual styles (e.g., innovation-seeking thinking style) are linked to both adaptive (e.g., the confrontative strategy and the planful problem-solving strategy) and maladaptive coping strategies (e.g., the escape-avoidance strategy). Relationships between Type III and coping strategies are also revealed in the previous literature. The external style is related to adaptive coping strategies, and the internal style is associated with maladaptive coping strategies (Gras et al., 2009). Therefore, it is worthwhile to further explore the heuristic value of intellectual styles to coping strategies among postgraduate students in Hong Kong.

## The Present Study

This study examined the influential roles of demographic factors (e.g., age, gender, social role orientation, marital status, and academic disciplines) in thinking styles and the predictive role of thinking styles for coping strategies with the control of demographic factors among mainland postgraduate students. This study is significant because it can enrich empirical knowledge of both relationships of the two aforementioned constructs, respectively. In addition, this study is also helpful to help postgraduate students to deal with coping strategies by advantage of thinking styles and their demographic factors.

To have a comprehensive understanding of the predictive power of thinking styles for coping strategies, all 13 thinking styles were examined in the present study. Coping strategies were categorized into three types as indicated in the literature review. The relationships were hypothesized as listed:

Hypotheses 1: It was predicted that most non-traditional-aged, men, masculine, married participants with higher socioeconomic status would tend to show Type I thinking styles, and the majority of traditional-aged, women, feminine, unmarried participants with lower socioeconomic status would exhibit Type II thinking styles.
Hypotheses 2: Students of natural science would prefer the global (I), executive (II) and, internal (III) styles, and students of social sciences would tend to deal with tasks with the monarchic (II) and external (III) styles. Finally, students of humanities would prefer conservative (II), internal (III), and oligarchic styles (III). No hypotheses were made on liberal, conservative, legislative, judicial, hierarchical, anarchic, and local styles.
Hypotheses 3: It was predicted that the thinking styles of participants would be associated with coping strategies. To be specific, Type I thinking styles would be positively linked to adaptive self-directed coping strategies, while Type II thinking styles would be positively associated with maladaptive other-directed coping and relinquished control coping strategies. At the same time, Type I and Type II thinking styles would be positively related to other-directed coping strategies. Type III thinking styles would be related to all coping strategies.

## Method

### Participants

A total of 148 postgraduate students (112 women and 36 men) were recruited from a university in Hong Kong. This study was based on a simple random sampling approach for conscious and unbiased purposes (Kirk, 2011). The ages of participants ranged from 22 to 42 years, with the mean and the median age being 30 years. Of the participants, 86 (58.1%) were traditional-aged and 62 (41.9%) were non-traditional-aged. Ethical clearance was endorsed by the Human Research Ethics Committee before collecting data. Before the questionnaires, participants were required to complete consent forms, which informed that their participation was entirely voluntary, and they had the right to withdraw from the research without any negative consequences at any time. Among the participants, 35.9% (*N* = 53) were in natural sciences, 39.9% (*N* = 59) in social sciences, and 24.3% (*N* = 36) in arts and humanities. Of the participants, 83.1% (*N* = 123) were unmarried, and the rest of the participants were married. Concerning their socioeconomic status, 22.3% (*N* = 33) of postgraduate students were in low, 53.4% (*N* = 79) in middle, and 24.3% (*N* = 36) in high socioeconomic status. In terms of the gender-role orientations of participants, 3.4% (*N* = 5) were masculine, 9.5% (*N* = 14) were feminine, 85.5% (*N* = 127) were androgynous, and 1.4% (*N* = 2) were undifferentiated. Personal reports were given in appreciation for their participation. The detailed distribution of the sample is presented in Table 1.

**Table 1:** The detailed distribution of the sample.

		**Number**
Age	Traditional age (22–25)	86
	Nontraditional age (>25)	62
Gender	Male	36
	Female	112
Gender role orientation	Masculinity	5
	Femininity	14
	Androgyny	127
	Undifferentiation	2
Marital status	Unmarried	123
	Married	25
Socioeconomic status	Low	33
	Middle	79
	High	36
Academic disciplines	Natural Science	53
	Social Science	59
	Arts and Humanities	36

### Measures

Participants responded to the aforementioned demographic information, TSI-R2 (Sternberg et al., 2007), and *COPE Revised* (Yuan et al., 2017).

#### Thinking Styles

The TSI-R2 (Sternberg et al., 2007), on the basis of Sternberg's theory of mental self-government, is employed to measure the thinking styles of the participants. It is a non-timed self-report questionnaire of 65 statements in five groups, and each subscale of thinking styles is assessed by five statements (Sternberg et al., 2007). Respondents complete the questionnaire and rate themselves on a 7-point Likert scale, with “1” representing that the statement does not describe themselves at all, and “7” showing that the statements describe themselves very well (Zhang et al., 2006). The origin version of the TSI-R2 is in English, and a translated and back-translated traditional Chinese version (Zhang, 2004) was used in this study since the majority of participants were mainland postgraduate students. The TSI-R2 (Sternberg et al., 2007) is the updated version of the TSI-R (Sternberg et al., 2003), which is used to further improve the internal consistency for the anarchic style. In this version of the anarchic style, the Cronbach's alpha coefficients for TSI-R2 indicate satisfactory internal reliability above 0.60 (Nunnally, 1978), and the internal reliability of all the other thinking styles range from 0.63 to 0.86.

#### COPE Revised

Coping strategies are measured by the COPE Revised (Yuan et al., 2017), which is a short version derived from Carver's Brief COPE (1997) for the sake of both brevity and sound psychometric properties. The Brief COPE (Carver, 1997), a short version of COPE (Carver et al., 1989), aims at assessing thirteen types of coping strategies (Skinner et al., 2003) and has been widely applied in multiple settings. Generally, the Brief COPE is a sound psychometric measurement since the Cronbach's alpha coefficients for most scales indicate satisfactory internal reliabilities above 0.60 (Nunnally, 1978). Nevertheless, low internal consistency has also been found in previous studies (Doron et al., 2011). At the same time, factor analysis also revealed the fragments of subscales. Thus, the Brief COPE (Carver, 1997) sacrifices satisfactory psychometric properties for brevity. To keep a balance of both psychometric properties and brevity, the COPE Revised, also called the three-dimensional coping strategy model (Yuan et al., 2017), is developed to assess coping strategies, which is composed of 45 items with 15 three-item subscales and adopt Seven-point Likert scale with “1” indicating the least frequent employment of the strategy in the statement and “7” showing the most prevalent employment of the strategy in the statement. Cronbach's alpha coefficients of the COPE Revised are ranging from 0.82 to 0.83 after the modification, and the validity has also been tested by exploratory factor analysis and confirmatory factor analysis (Yuan et al., 2017).

### Data Analysis

Firstly, reliabilities of *TSI-R2* (Sternberg et al., 2007) and *COPE Revised* (Yuan et al., 2017) were examined. The internal reliability of these two inventories was estimated by Cronbach's alpha (1951). Secondly, multivariate ANOVA (MANOVA) was conducted to statistically examine the influence of demographic factors (i.e., age, gender, social-role orientation, socioeconomic status, marital status, and academic disciplines) on thinking styles. Finally, hierarchical multiple regression analyses with the stepwise method were also be utilized to statistically explore the predictive power of thinking styles in coping strategies beyond the influence of demographic factors.

## Results

### Internal Scale Reliability of TSI-R2 and COPE Revised

The Cronbach's alpha coefficients for the TSI-R2 scales were 0.71 (legislative), 0.63 (executive), 0.76 (judicial), 0.73 (global), 0.73 (local), 0.84 (liberal), 0.75 (conservative), 0.72 (hierarchical), 0.72 (monarchic), 0.75 (oligarchic), 0.66 (anarchic), 0.79 (internal), and 0.78 (external). These figures are consistent with those obtained in previous studies among secondary school students and university students in Hong Kong and mainland China (Zhang et al., 2013; Yuan et al., 2017). The reliability was reasonably good for TSI-R2.

The *COPE Revised* (Yuan et al., 2017) exhibited acceptable reliability with all scales exceeding 0.50 (Nunnally, 1978) and, the majority of scales (except for active coping and venting) were in their mid 0.70s. The three-dimensional hierarchical model (Yuan et al., 2017) also exhibited good Cronbach alpha coefficients, and they were 0.86, 0.87, and 0.79 for the self-directed dimension, the other-directed dimension and the relinquished control dimension separately. Correlations among key variables and statistics from hierarchical multiple regressions are presented in Tables 2 and 3.

**Table 2:** Influential roles of demographic factors on thinking styles.

**Source**	**Dimensions**	**Sum of squares**	***df***	**Mean Square**	**F**	**Sig**.	**Partial Eta squared**
Gender	Internal	4.99	1	4.99	4.61	0.034	0.030
Gender role orientation	Judicial	8.54	3	2.85	3.23	0.024	0.063
	Liberal	18.22	3	6.07	5.54	0.001	0.103
	Internal	8.73	3	2.91	2.71	0.047	0.053
Age	Local	4.95	1	4.95	4.19	0.042	0.028
	Hierarchical	5.48	1	5.48	7.98	0.005	0.052
	Anarchic	4.08	1	4.08	3.92	0.050	0.026
Marital status	Monarchic	5.49	1	5.49	5.54	0.020	0.037
	Oligarchic	4.51	1	4.51	4.347	0.039	0.029

**Table 3:** Predicting coping strategies from thinking styles.

**SCS**	**Self-directed coping**		**Other-directed coping**				**Relinquished control coping**			
	**Active coping**	**Planning**	**Positive rei**	**Emotional support**	**Instrumental support**	**Self-distraction**	**Venting**	**Acceptance**	**Self-blame**	**Social withdrawal**
R^2^_Total_	0.25	0.37	0.38	0.07	0.24	0.21	0.20	0.16	0.16	0.18
R^2^_demo_	0.03	0.08	0.03	0.00	0.03	0.03	0.07	0.00	0.00	0.00
R^2^_TS_	0.22	0.29	0.35	0.07	0.21	0.18	0.13	0.16	0.16	0.18
F	11.67***	16.17***	12.29***	10.30**	11.31***	9.51***	9.05***	13.27***	10.31***	10.37***
df	147	147	147	147	147	147	147	147	147	147
β_leg_						0.21**				
β_lib_	0.16*		0.30***							
β_hie_	0.30**	0.41***					0.27**			−0.24**
β_exe_					0.21*					
β_loc_									0.24**	0.15*
β_con_								0.33***		
β_oli_					−0.28**	0.20*				
β_ana_		−0.15*				0.26**		0.18*	0.20*	
β_int_										0.35***
β_ext_	0.19*	0.21**		0.26**	0.43***		0.22**			

*CS, coping strategies; R^2^_Total_, the contribution of gender, social-role orientation, age, marital status, socioeconomic status, and academic disciplines to thinking styles; R^2^_TS_, the unique contribution of thinking styles to coping strategies; positive rei, positive reinterpretation; leg, legislative; lib, liberal; hie, hierarchical; exe, executive; loc, local; con, conservative; oli, oligarchic; ana, anarchic; int, internal; ext, external*;

* *p < 0.05*.

** *p < 0.01*.

*** *p < 0.001*.

### Influential Roles of Demographic Factors on Thinking Styles

Firstly, findings from the MANOVA showed that male participants were perceived to behave more preference for internal styles than female participants (mean_male_ = 5.08, mean_female_ = 4.66, *t* = 2.15, *p* < 0.05). Secondly, undifferentiated participants scored the highest in judicial (mean_masculinity_ = 4.04, mean_femininity_ = 4.49, mean_androgyny_ = 5.05, mean_undifferentiation_ = 5.30, *p* < 0.05), and liberal (mean_masculinity_ = 3.72, mean_femininity_ = 3.47, mean_androgyny_ = 4.58, mean_undifferentiation_ = 4.70, *p* = 0.001) styles. Also, androgynous participants tended to perform internal styles (mean_masculinity_ = 5.00, mean_femininity_ = 4.01, mean_androgyny_ = 4.83, mean_undifferentiation_ = 4.80, *p* < 0.05) more in dealing with tasks than other three types of participants. Thirdly, traditional-aged participants exhibited more local (mean_traditional−aged_ = 4.24, mean_nontraditional−aged_ = 3.87, *t* = 2.05, *p* < 0.05), less hierarchical (mean_traditional−aged_ = 5.14, mean_nontraditional−aged_ = 5.53, *t* = −2.82, *p* < 0.01), and more anarchic styles (mean_traditional−aged_ = 4.08, mean_nontraditional−aged_ = 3.74, *t* = 1.98, *p* = 0.05) to deal with tasks than non-traditional-aged participants.

Results from the *t*-test showed that unmarried participants had less preference for using the monarchic style (mean_unmarried_ = 4.86, mean_married_ = 5.38, *t* = −2.36, *p* < 0.05) and the oligarchic style (mean_unmarried_ = 4.60, mean_married_ = 5.06, *t* = −2.09, *p* < 0.05). No statistical differences have been found in the relationships between academic disciplines and thinking styles and the relationships between socioeconomic status and thinking styles.

### Predicting Coping Strategies From Thinking Styles With Demographic Factors Controlled

With relevant demographic factors being taken into consideration, results from hierarchical multiple regressions indicated that 10 thinking styles significantly contributed to all 10 coping strategies. These unique contributions of thinking styles to coping strategies ranged from 7 to 35%. From the statistical perspective, all contributions are in the expected directions. First, two Type I (liberal and hierarchical) and two Type II styles (external and anarchic) accounted for 34.9 % of the observed variance in self-directed coping strategies. The contribution of two Type I styles and one Type III style (external) was positive, while that of the anarchic style was negative. Second, one Type I style (hierarchical), two Type II styles (local and conservative), and two Type III styles (anarchic and internal) accounted for 34.2% of the observed variance in relinquished control coping strategies. Two Type II styles (local and conservative) and two Type III styles (anarchic and internal) were significantly positively predicted, whereas one Type I style (hierarchical) was significantly negatively predicted. Third, two Type I styles (legislative and hierarchical), one Type II style (executive), and three Type III styles (oligarchic, anarchic, and external) accounted for 7.9% of the observed variance in other-directed coping strategies. The contribution of two Type I styles (legislative and hierarchical), one Type II style (executive), and two Type III styles (anarchic and external) positively predicted other-directed coping strategies, whereas one Type II style (oligarchic) both positively predicted one other-directed coping strategy (self-distraction) and negatively predicted another other-directed coping strategy (instrumental support). Hence, as was anticipated based on the theoretical framework and empirical evidence, Type I styles (liberal and hierarchical) significantly positively predicted self-directed coping strategies, and Type II styles (local and conservative) significantly positively predicted relinquished control strategies.

## Discussion

The first primary objective concerned the influential roles of demographic factors on thinking styles. In the beginning, two inventories were validated among mainland postgraduate students in Hong Kong. Results demonstrated that non-traditional-aged participants exhibited less local, more hierarchical, and less anarchic styles to deal with tasks than traditional-aged participants, which are consistent with the hypotheses. Compared to traditional-aged participants, non-traditional-aged participants tended to have more working experiences, in which they were exposed to more complicated multi-task environments with various challenges and difficulties. Thus, they have to nurture a higher level of cognitive complexity, such as dealing with tasks in an orderly way or making decisions globally to cope with their work efficiently. Thus, it is understandable that non-traditional-aged postgraduate students have more preferences for Type I thinking styles. This finding can lend support to previous studies (Zhang and He, 2011; Kuan and Zhang, 2020).

Gender difference has no significant influence on Type I and Type II thinking styles. The findings are contradicted the hypotheses and previous studies among Hong Kong university students, Hong Kong secondary students, and mainland Chinese secondary students (Zhang and Sachs, 1997; Cheung, 2002; Zhang, 2003). Previous studies showed that men were generally associated with Type I thinking styles, and women were frequently related to Type II thinking styles. The discrepancy could be partially accounted for by different social expectations and different methods of nurture. However, the insignificant effects of gender on thinking styles could also be confounded by the function of socialization, such as their educational experiences in the period of postgraduate studies. Postgraduate students need to tackle multiple tasks. Thus, they have to sufficiently finish different tasks in an orderly way. If male or female postgraduate students want to get high grades, they need to show critical and innovative ideas in their assignments. These ideas encourage students to promote Type I thinking styles. Thus, no gender difference could be seen as a result of the socialization of postgraduate students' learning requirements or learning objectives. Another explanation is that the standard of admission to the master's programs is related to Type I thinking styles. That is to say, the majority of students who are able to get admitted into postgraduate studies already possess Type I thinking styles, thus no significant gender difference in the relationship of thinking styles could be perceived in the current study. Moreover, the present study found that one Type III thinking style (internal) was positively associated with male postgraduate students. This suggests that male postgraduate students tend to work alone on a task and make decisions relying on their own judgment.

No previous literature has reviewed the relationship between gender-role orientation and thinking styles. As shown in the results section, undifferentiated participants scored the highest in the judicial and the liberal style. The positive effect of undifferentiation on Type I thinking styles was surprising and has not yet been found in previous studies. It may be because previous hypotheses only consider masculinity and the femininity regardless of undifferentiation and androgyny as key variables of gender-role orientations to examine the relationship with thinking styles (Bem and Lenney, 1976; Zhang, 2013). More surprisingly, undifferentiation is always considered to be associated with poorer socialization, limited behavioral flexibility (Bem, 1974), low self-esteem (Chow, 1987), and little cognitive complexity in evaluating careers (Harren, 1979). However, in the present study, undifferentiation was positively linked to superior Type I thinking styles. Thus, the function of undifferentiation is needs to be delved into in further research. Besides, the result showed that androgynous participants also tended to perform internal styles more in dealing with tasks than the other three types of participants. This means that androgynous participants prefer to work alone and make their own decisions based on their judgment.

The finding showed that unmarried participants have less preference for using monarchic and oligarchic styles, which was also contradicted the hypotheses. If married participants continue to further improve themselves in postgraduate study, they may decrease the time available to spend with their family to a certain extent. Thus, under invisible economic and psychological pressure, this kind of participant could probably spend most of their energy on their academic work to graduate on time. Therefore, married participants were associated with the monarchic thinking style. Furthermore, those postgraduate students who have got married may tend to consider their families before making decisions. Thus, married postgraduate students prefer to possess the oligarchic style.

Results showed that neither academic disciplines nor socioeconomic status have a significant influence on thinking styles. This finding was also surprising and contradictary to previous findings. This may be explained by the small sample size in the present research. Another explanation can also be accounted for the requirements of admission and learning objectives. As aforementioned, those who possess Type I thinking styles might be more likely to get admitted into postgraduate studies regardless of their academic disciplines and socioeconomic status. Furthermore, the learning requirements encompassing all academic disciplines may push students to promote their Type I thinking styles (the legislative, judicial, liberal, and global styles).

The second objective concerned the predictive roles of thinking styles for coping strategies. Results demonstrated that Type I thinking styles positively contributed to self-directed coping strategies and Type II thinking styles positively played the predictive roles for relinquished control coping strategies, supporting the hypothesis of this study. Despite the maximum variance in coping strategies explained by thinking styles being about 35% among participants, the predictive roles of thinking styles for coping strategies are more likely to reflect true variations rather than have been found by chance, for three reasons. First, the general pattern is that students with Type I thinking styles prefer to utilize self-directed coping strategies, and students with Type II thinking styles tend to employ relinquished control coping strategies. Given stressors and challenges students experience in universities, students are more likely to consider assigning and dealing with priorities in a novel and orderly way. Hence, they would be more efficient and comfortable in developing different solutions, making plans, and giving priorities to the significant stressors. Similarly, when facing stress and difficulties, students of Type II thinking styles might accept the stress negatively, blame themselves, and stay away from their friends and family. Type II thinking style (executive) was also found to be positively associated with other-directed coping (the use of instrumental support). It is natural for students who get used to following specific rules or directions tend to get advice from experienced people.

Second, the use of other-directed coping strategies is in association with the preference of both Type I and Type II thinking styles. When confronting stressful situations, students with Type I thinking styles have a preference for using their own strategies and ideas to relieve their stress by self-dependent activities such as going to movies, reading, sleeping, or listening to music to distract their attention. It is natural that students with the executive style (one Type II style) would prefer to ask for and execute suggestions when dealing with stressors. Third, the relationships that predictive roles of Type III thinking styles in all three types of coping strategies make substantial sense. A preference for the anarchic style was negatively associated with self-directed coping strategies and positively related to relinquished control coping strategies. It could be interpreted that students who tended to tackle different stressors affecting them with equal urgency ended up feeling helpless and desperat trying to handle all the stressors at once and thus were more likely to eventually give up (relinquished control coping strategies). While the external style was positively associated with self-directed and other-directed coping, the internal style was positively associated with the relinquished control coping strategies. If students attempted to share their stress situations with friends or classmates they thus might have more opportunities to collect more information on how to deal with that stress, or receive suggestions from their friends.

## Contributions, Implications, and Limitations

This study has made three contributions. Firstly, two inventories [i.e., TSI-R2 (Sternberg et al., 2007) and *COPE Revised* (Yuan et al., 2017)] have been validated in the context of Hong Kong. Secondly, the present study showed that thinking styles have a unique predictive power in coping strategies. Furthermore, it also pioneered the relationships between thinking styles and gender-role orientations, which depicts a more dynamic picture of the relationships between thinking styles and key demographic variables. Thirdly, empirical evidence has been provided to support the theory of Zhang and Sternberg's intellectual styles (Zhang and Sternberg, 2005) and other theories. For example, the research showed the predictive roles of thinking styles in coping strategies, which supports the argument of the value of intellectual styles. Furthermore, findings of the present research lend support to the gender-role orientation by providing empirical evidence on the discussion of the function of androgyny to thinking styles.

Significant implications provide insight into educational and psychological practice. The finding has proved the value of thinking styles by examining the relationship with coping strategies. In other words, Type I thinking styles have been identified as adaptive thinking styles, which provides valuable information for postgraduate students, academics, and administrators in higher institutional backgrounds. According to the findings in the present research, creativity-generating Type I thinking styles are more likely to help postgraduate students to utilize adaptive coping strategies (self-directed coping strategies). Norm-conforming Type II thinking styles seem to be related to the maladaptive coping strategies (relinquished control coping strategies). In other words, Type I thinking styles are more beneficial for student development. Bearing this in mind is of significance in psychological and educational settings. For postgraduate students, they can search for opportunities to cultivate Type I thinking styles in both their academic performance and their daily lives. For example, when handling multiple tasks with great pressure, postgraduate students are advised to think of their own innovative ideas or creative strategies to analyze and deal with stressful situations. For academics aiming at promoting Type I thinking styles, academics should give students the maximum freedom to invent their own ideas and share their ideas with other students, which provide opportunities to inspire others. For administrators, they should arrange their curriculums considering innovative and critical thinking as their standards of assessment.

Although we foresee significant contributions by this research, three main limitations should be pointed out. Firstly, data in the quantitative research elucidated self-reported inventories require retrospection, which might influence objectiveness. Secondly, the population of the sample is not large enough and only is conducted in the Hong Kong context, which might not be over-generalized to represent other student populations in other areas. Thirdly, participants might withdraw from the search due to the long period of time required to finish the questionnaire, which might influence the specific demographic factors or disciplines representing the particular populations.

## Data Availability Statement

The raw data supporting the conclusions of this article will be made available by the authors, without undue reservation.

## Ethics Statement

The studies involving human participants were reviewed and approved by the Human Research Ethics Committee, HKU. Written informed consent for participation was not required for this study in accordance with the national legislation and the institutional requirements.

## Author Contributions

SC contributed to conception and design of the study, organized the database, performed the statistical analysis, wrote the first draft of the manuscript, wrote sections of the manuscript, manuscript revision, read, and approved the submitted version.

## Conflict of Interest

The author declares that the research was conducted in the absence of any commercial or financial relationships that could be construed as a potential conflict of interest.

## Publisher's Note

All claims expressed in this article are solely those of the authors and do not necessarily represent those of their affiliated organizations, or those of the publisher, the editors and the reviewers. Any product that may be evaluated in this article, or claim that may be made by its manufacturer, is not guaranteed or endorsed by the publisher.

## References

[B1] Adorno T. Frenkel-Brenswik E. Levinson D. J. Sanford R. N. (2019). The Authoritarian Personality. Cham: Verso Books.

[B2] Alevriadou A. Hatzinikolaou K. Tsakiridou H. Grouios G. (2004). Field dependence-independence of normally developing and mentally retarded boys of low and upper/middle socioeconomic status. Percept. Motor Skills 99, 913–923. 10.2466/pms.99.3.913-923 15648488

[B3] Allport G. W. (1937). Personality: A Psychological Interpretation. Holt.

[B4] Al-Thani A. Al-Thani T. Semmar Y. (2014). Investigating the relationship between students' thinking styles, self-efficacy for learning, and academic performance at Qatar University. Am. Int. J. Soc. Sci. 3, 172–179.

[B5] Appelhans B. M. Schmeck R. R. (2002). Learning styles and approach versus. avoidant coping during academic exam preparation. Coll. Student J. 36, 157–161.

[B6] Armor D. A. Taylor S. E. (2003). The effects of mindset on behavior: self-regulation in deliberative and implemental frames of mind. Pers. Soc. Psychol. Bull. 29, 86–95. 10.1177/0146167202238374 15272962

[B7] Balkis M. Isiker G. B. (2005). The relationship between thinking styles and personality types. Soc. Behav. Pers. Int. J. 33, 283–294. 10.2224/sbp.2005.33.3.283

[B8] Becher T. (1981). Towards a definition of disciplinary cultures. Stud. Higher Educ. 6, 109–122. 10.1080/0307507811233137936233302463

[B9] Bem S. L. (1974). The measurement of psychological androgyny. J. Consult. Clin. Psychol. 42:155. 10.1037/h0036215 4823550

[B10] Bem S. L. Lenney E. (1976). Sex typing and the avoidance of cross-sex behavior. J. Pers. Soc. Psychol. 33:48. 10.1037/h0078640 1018227

[B11] Bhowmik M. K. Cheung R. Y. Hue M. T. (2018). Acculturative stress and coping strategies among Mainland Chinese university students in Hong Kong: a qualitative inquiry. Am. J. Orthopsychiatry 88:550. 10.1037/ort0000338 30179027

[B12] Billings A. G. Moos R. H. (1984). Coping, stress, and social resources among adults with unipolar depression. J. Pers. Soc. Psychol. 46:877. 10.1037/0022-3514.46.4.877 6737198

[B13] Bishop C. Foster C. (2011). Thinking styles: maximizing online supported learning. J. Educ. Comput. Res. 44, 121–139. 10.2190/EC.44.2.a

[B14] Broberg G. C. Moran J. D. III (1988). Creative potential and conceptual tempo in preschool children. Creat. Res. J. 1, 115–121. 10.1080/10400418809534293

[B15] Brown L. Holloway I. (2008). The adjustment journey of international postgraduate students at an English university: an ethnographic study. J. Res. Int. Educ. 7, 232–249. 10.1177/1475240908091306

[B16] Carlson R. Levy N. (1968). Brief method for assessing social-personal orientation. Psychol. Rep. 23, 911–914. 10.2466/pr0.1968.23.3.911 5717461

[B17] Carver C. S. (1997). You want to measure coping but your protocol's too long: consider the brief COPE. Int. J. Behav. Med. 4, 92–100. 10.1207/s15327558ijbm0401_6 16250744

[B18] Carver C. S. Scheier M. F. Weintraub J. K. (1989). Assessing coping strategies: a theoretically based approach. J. Pers. Soc. Psychol. 56:267. 10.1037/0022-3514.56.2.267 2926629

[B19] Cheung E. (2002). Students' Thinking Styles, Learning Approaches, and Instructional Preferences: Their Relationships With Academic Achievement in Different Disciplines. Unpublished manuscript. The University of Hong Kong.

[B20] Chow E. N. L. (1987). The influence of sex-role identity and occupational attainment on the psychological well-being of Asian American women. Psychol. Women Q. 11, 69–082. 10.1111/j.1471-6402.1987.tb00775.x

[B21] Coffield F. C. Moseley D. V. M. Hall E. Ecclestone K. (2004). Learning Styles and Pedagogy in Post-16 Learning: Findings of a Systematic and Critical Review of Learning Styles Models. London: Learning and Skills Research Center.

[B22] Compas B. E. Connor J. Osowiecki D. Welch A. (1997). “Effortful and involuntary responses to stress,” in Coping with Chronic Stress (Boston, MA: Springer), 105–130. 10.1007/978-1-4757-9862-3_4

[B23] Cronbach L. J. (1951). Coefficient alpha and the internal structure of tests. Psychometrika 16, 297–334. 10.1007/BF02310555

[B24] Curry L. (1983). An Organization of Learning Styles Theory and Constructs. Available online at: https://files.eric.ed.gov/fulltext/ED235185.pdf

[B25] Demes K. A. Geeraert N. (2015). The highs and lows of a cultural transition: a longitudinal analysis of sojourner stress and adaptation across 50 countries. J. Pers. Soc. Psychol. 109:316. 10.1037/pspp0000046 26191963 PMC4507515

[B26] Doron J. Stephan Y. Maiano C. Le Scanff C. (2011). Motivational predictors of coping with academic examination. J. Soc. Psychol. 151, 87–104. 10.1080/00224540903366768 21375127

[B27] Fan J. (2016). The role of thinking styles in career decision-making self-efficacy among university students. Think. Skills Creat. 20, 63–73. 10.1016/j.tsc.2016.03.001

[B28] Fan W. (2012). An experimental comparison of the flexibility in the use of thinking styles in traditional and hypermedia learning environments. Think. Ski. Creat. 7, 224–233.

[B29] Fer S. (2007). What are the thinking styles of Turkish student teachers? Teach. Coll. Rec. 109, 1488–1516.

[B30] Folkman S. Lazarus R. S. (1980). An analysis of coping in a middle-aged community sample. J. Health Soc. Behav. 219–239. 10.2307/2136617 7410799

[B31] Folkman S. Lazarus R. S. (1984). Stress, Appraisal, and Coping. New York, NY: Springer Publishing Company.

[B32] Gras R. M. L. Berná J. C. López P. S. (2009). Thinking styles and coping when caring for a child with severe spina bifida. J. Dev. Phys. Disabil. 21:169. 10.1007/s10882-009-9133-0

[B33] Gridley M. C. (2006). Thinking styles in a sample of women engineers. Psychol. Rep. 98, 911–914. 10.2466/pr0.98.3.911-914 16933695

[B34] Grigorenko E. L. Sternberg R. J. (1995). “Thinking styles,” in International Handbook of Personality and Intelligence (Boston, MA: Springer), 205–229. 10.1007/978-1-4757-5571-8_11

[B35] Grigorenko E. L. Sternberg R. J. (1997). Styles of thinking, abilities, and academic performance. Except. Child. 63, 295–312. 10.1177/001440299706300301

[B36] Guisande M. A. Páramo M. F. Tinajero C. Almeida L. S. (2007). Field dependence-independence (FDI) cognitive style: an analysis of attentional functioning. Psicothema 19, 572–577. 17959109

[B37] Han J. Chu X. Song H. Li Y. (2014). Social capital, socioeconomic status and self-efficacy. Appl. Econ. Fin. 2, 1–10. 10.11114/aef.v2i1.607

[B38] Harren V. A. (1979). A model of career decision making for college students. J. Voc. Behav. 14, 119–133. 10.1016/0001-8791(79)90065-4

[B39] Ho M. C. (2011). Academic discourse socialization through small-group discusssions. System 39, 437–450 10.1016/j.system.2011.10.015

[B40] Hofstede G. H. (1980). Culture's Consequences: International Differences in Work-Related Values. Beverly Hills, CA: Sage.

[B41] Huston A. C. (1985). The development of sex typing: themes from recent research. Dev. Rev. 5, 1–17. 10.1016/0273-2297(85)90028-0

[B42] Jain D. Tiwari G. K. Awasthi I. (2018). Average level of socioeconomic status is conducive for metacognitive awareness and academic success. Madhya Bharti 74, 207–221.

[B43] Joniak A. J. Isaksen S. G. (1988). The Gregorc Style Delineator: Internal consistency and its relationship to Kirton's adaptive-innovative distinction. Educ. Psychol. Meas. 48, 1043–1049.

[B44] Karagiannopoulou E. Christodoulides P. (2005). The impact of Greek University students' perceptions of their learning environment on approaches to studying and academic outcomes. Int. J. Educ. Res. 43, 329–350. 10.1016/j.ijer.2006.05.002

[B45] Keller R. T. Holland W. E. (1978). A cross-validation study of the Kirton Adaption-Innovation Inventory in three research and development organizations. Appl. Psychol. Meas. 2, 563–570.

[B46] Kim M. (2010). The relationship between thinking style differences and career choice for high-achieving high school students. Dissert. Abstracts Int. A Human. Soc. Sci. 70:2902.

[B47] Kirk M. (2011). “Simple random sample,” in International Encyclopedia of Statistics Science, ed M. Lovric (Berlin: Springer), 1328–1330. 10.1007/978-3-642-04898-2_518

[B48] Kuan T. Y. J. Zhang L. F. (2020). Thinking styles and time perspectives. Educ. Psychol. 1–19. 10.1080/01443410.2020.1730306

[B49] Lam P. Y. (2000). The Usefulness of Thinking Styles in Reflecting How Individuals Think and Explaining School Performance. Unpublished manuscript. The University of Hong Kong.

[B50] Lau C. H. (2014). Thinking Styles, Motivational Orientations, and Academic Achievement in Learning Physics Among Hong Kong Secondary School Students. Hong Kong: University of Hong Kong Libraries.

[B51] Lue B. Chen H. Wang C. Cheng Y. Chen M. (2010). Stress, personal characteristics and burnout among first postgraduate year residents: A nationwide study in Taiwan. Medical Teacher 32, 400–407. 20423259 10.3109/01421590903437188

[B52] Maccoby E. E. Jacklin C. N. (1974). Myth, reality and shades of gray-what we know and don't know about sex differences. Psychol. Today 8, 109–112. 10.1037/e400662009-008

[B53] McCrae R. R. (1982). Consensual validation of personality traits: evidence from self-reports and ratings. J. Pers. Soc. Psychol. 43:293. 10.1037/0022-3514.43.2.29315907164

[B54] Messick S. (1994). The matter of style: manifestations of personality in cognition, learning, and teaching. Educ. Psychol. 29, 121–136.

[B55] Miller A. (1987). Cognitive styles: an integrated model. Educ. Psychol. 7, 251–268.

[B56] Murphy A. Janeke H. C. (2009). The relationship between thinking styles and emotional intelligence: an exploratory study. South Afr. J. Psychol. 39, 357–375. 10.1177/008124630903900310

[B57] Nash S. C. (1979). “Sex role as a mediator of intellectual functioning,” in Sex-Related Differences in Cognitive Functioning: Developmental Issues, eds M. A. Wittig and A. C. Peterson (New York, NY: Academic Press), 263–302.

[B58] Nunnally J. C. (1978). Psychometric Theory, 2nd Edn. McGraw-Hill series in Psychology. New York, NY: McGraw-Hill.

[B59] Pettigrew A. C. King M. O. (1993). A comparison between scores on Kirton's inventory for nursing students and a general student population. Psychol. Rep. 73, 339–345. 10.2466/pr0.1993.73.1.339 8367575

[B60] Pinker S. (1997). How the Mind Works. New York, NY: W. W. Norton and Company.

[B61] Powers A. S. Myers J. E. Tingle L. R. Powers J. C. (2004). Wellness, perceived stress, mattering, and marital satisfaction among medical residents and their spouses: implications for education and counseling. Family J. 12, 26–36. 10.1177/1066480703258802

[B62] Poyrazli S. Kavanaugh P. R. (2006). Marital status, ethnicity, academic achievement, and adjustment strains. Coll. Student J. 40, 767–780.

[B63] Ross J. (1962). “Factor analysis and levels of measurement in psychology,” in Measurement in Personality and Cognition, 69–81.

[B64] Roth S. Cohen L. J. (1986). Approach, avoidance, and coping with stress. Am. Psychol. 41, 813. 10.1037/0003-066X.41.7.813 3740641

[B65] Ryan-Wenger N. M. (1992). A taxonomy of children's coping strategies: a step toward theory development. Am. J. Orthopsychiatry 62, 256–263. 10.1037/h0079328 1580343

[B66] Skinner E. A. (1999). "Action regulation, coping, and development," in *Action* & *Self-Development: Theory and Research Through the Life Span*, eds J. Brandtstädter and R. M. Lerner (Washington, DC: Sage Publications), 465–503.

[B67] Skinner E. A. Edge K. Altman J. Sherwood H. (2003). Searching for the structure of coping: a review and critique of category systems for classifying ways of coping. Psychol. Bull. 129, 216–269. 10.1037/0033-2909.129.2.216 12696840

[B68] Sternberg R. J. (1988). Mental self-government: a theory of intellectual styles and their development. Hum. Dev. 31, 197–224. 10.1159/000275810

[B69] Sternberg R. J. (1997). Thinking Styles. New York, NY: Cambridge University Press. 10.1017/CBO9780511584152

[B70] Sternberg R. J. Wagner R. K. Zhang L. F. (2003). Thinking Styles Inventory Revised. Unpublished test, Yale University.

[B71] Sternberg R. J. Wagner R. K. Zhang L. F. (2007). Thinking Styles Inventory - Revised II. Tufts University. Unpublished test.

[B72] Stuart I. R. Breslow A. Brechner S. Ilyus R. B. Wolpoff M. (1965). The question of constitutional influence on perceptual style. Percept. Motor Skills 20, 419–420. 10.2466/pms.1965.20.2.419 14279313

[B73] Tolor A. Fehon D. (1987). Coping with stress: a study of male adolescents' coping strategies as related to adjustment. J. Adolesc. Res. 2, 33–42. 10.1177/074355488721003

[B74] Tummala-Narra P. Deshpande A. Kaur J. (2016). South Asian adolescents' experiences of acculturative stress and coping. Am. J. Orthopsychiatry 86:194. 10.1037/ort0000147 26765547

[B75] University Grants Committee (2020). General Statistics on UGC-Funded Institutions.

[B76] Vaught G. M. (1965). The relationship of role identification and ego strength to sex. Differences in the rod-and-frame test. J. Pers. 33, 271–283 10.1111/j.1467-6494.1965.tb01386.x 14299701

[B77] Watson M. McMahon M. Foxcroft C. Els C. (2010). Occupational aspirations of low socioeconomic Black South African children. J. Career Dev. 37, 717–734. 10.1177/0894845309359351

[B78] Weisz J. R. McCabe M. A. Dennig M. D. (1994). Primary and secondary control among children undergoing medical procedures: adjustment as a function of coping style. J. Consult. Clin. Psychol. 62:324. 10.1037/0022-006X.62.2.324 8201070

[B79] Witkin H. A. Berry J. W. (1975). Psychological differentiation in cross-cultural perspective. J. Cross Cult. Psychol. 6, 4–87. 10.1177/002202217500600102

[B80] Young S. (2005). Coping strategies used by adults with ADHD. Pers. Individ. Diff. 38, 809–816. 10.1016/j.paid.2004.06.005

[B81] Yu B. Mak A. S. Bodycott P. (2019). Psychological and academic adaptation of mainland Chinese students in Hong Kong universities. Stud. Higher Educ. 46, 1552–1564. 10.1080/03075079.2019.1693991

[B82] Yuan W. Zhang L. F. Fu M. (2017). Thinking styles and academic stress coping among Chinese secondary school students. Educ. Psychol. 37, 1015–1025. 10.1080/01443410.2017.128734329749425

[B83] Zarshenas L. Sharif F. Molazem Z. Khayyer M. Zare N. Ebadi A. (2014). Professional socialization in nursing: a qualitative content analysis. Iran. J. Nurs. Midwifery Res. 19:432. 25183987 PMC4145501

[B84] Zhang L. Sternberg R. (2006). The Nature of Intellectual Styles (Educational Psychology Series), 1st Edn. Mahwah, NJ: Lawrence Erlbaum Associates.

[B85] Zhang L. F. (2002). Thinking styles and cognitive development. J. Genet. Psychol. 163, 179–195. 12095088 10.1080/00221320209598676

[B86] Zhang L. F. (2003). Are parents' and children's thinking styles related? Psychol. Rep. 93, 617–630. 10.2466/pr0.2003.93.2.617 14650696

[B87] Zhang L. F. (2004). Learning approaches and career personality types: Biggs and Holland united. Pers. Individ. Diff. 37, 65–81 10.1016/j.paid.2003.08.027

[B88] Zhang L. F. (2010). Do thinking styles contribute to metacognition beyond self-rated abilities?. Educ. Psychol. 30, 481–494. 10.1080/01443411003659986

[B89] Zhang L. F. (2013). The Malleability of Intellectual Styles. New York, NY: Cambridge University Press. 10.1017/CBO9780511973055

[B90] Zhang L. F. (2017). The Value of Intellectual Styles. New York, NY: Cambridge University Press. 10.1017/9781316014561

[B91] Zhang L. F. He Y. F. (2011). Thinking styles and the Eriksonian stages. J. Adult Dev. 18, 8–17. 10.1007/s10804-010-9101-z

[B92] Zhang L. F. Higgins P. (2008). The predictive power of socialization variables for thinking styles among adults in the workplace. Learn. Individ. Diff. 18, 11–18. 10.1016/j.lindif.2007.03.002

[B93] Zhang L. F. Jing L. Z. (2014). Organizational commitment and teaching styles. Among academics in mainland China. Educ. Psychol. (Dorchester-on-Thames) 36, 415–430.

[B94] Zhang L. F. Postiglione G. A. (2001). Thinking styles, self-esteem, and socio-economic status. Pers. Individ. Diff. 31, 1333–1346. 10.1016/S0191-8869(00)00227-08378557

[B95] Zhang L. F. Sachs J. (1997). Assessing thinking styles in the theory of mental self-government: a Hong Kong validity study. Psychol. Rep. 81, 915–928. 10.2466/pr0.1997.81.3.915

[B96] Zhang L. F. Sternberg R. J. (2005). A threefold model of intellectual styles. Educ. Psychol. Rev. 17, 1–53. 10.1007/s10648-005-1635-434744878

[B97] Zhang L. F. Sternberg R. J. Fan J. (2013). Revisiting the concept of 'style match'. Br. J. Educ. 83, 225–237. 23692532 10.1111/bjep.12011

[B98] Zhang L. F. Sternberg R. J. Sternberg R. J. (2006). The Nature of Intellectual Styles (Educational psychology series). Mahwah, NJ: Lawrence Erlbaum Associates.

